# 

**Published:** 1875-09

**Authors:** 


### CONTENTS:

### ORIGINAL COMMUNICATIONS.

ARTICLE I.—The Causes of Irregularity in the Development of the Teeth. (Concluded.) By N. W. Kingsley, D. D. S., M. D ........ 193

ARTICLE II.—How far are we Justified in Anticipating Proximate Decay. By E. D. Swain, D. D. S ........ 210

### SELECTED ARTICLES.

ARTICLE III.—Nitrous Oxide Gas. By H. J. Barnes, M. D ........ 217

ARTICLE IV.—The Treatment of Exposed Pulps in Filling Teeth. By Samuel Welchens, D. D. S ........ 221

ARTICLE V.—Treatment of a Fractured Tooth. By Theo. G. Lewis, D. D. S ........ 225

ARTICLE VI—A New Splint for Fractured Jaw. By Francis H. Atkins, M. D ........ 228

### EDITORIAL, ETC.

The Late Meeting at Long Branch ........ 231

The American Academy of Dental Surgery ........ 233

### OBITUARY.

Dr. R. M. Gage ........ 233

### MONTHLY SUMMARY.

Death from Ether Inhalation ........ 235

Poisoning by Aconite ........ 235

Carbolic Acid as an Antiphlositic ........ 236

Physiological Experiment Upon the Human Corpse ........ 236

Toothache ........ 236

Location of the Sense of Taste ........ 236

Efficiency of Mineral Water in Reducing and Adding Fat ........ 237

A New Cause for Blue Line on Gums ........ 237

Wine Drinking and Drunkenness ........ 237

A New Mode of Burial ........ 237

Over-Work or Over-Play ........ 238

A Calculus of Steno's Duct Having for its Origin a Grain of Wheat ........ 239

A Dangerous Compound ........ 239

A British Dental Association ........ 239

An Advance in Dental Surgery ........ 240

A Large Mouth ........ 240

A Cure for Drunkenness ........ 240

